# Psychosocial Factors Influencing Resilience in a Sample of Victims of Armed Conflict in Colombia: A Quantitative Study

**DOI:** 10.3390/bs15060816

**Published:** 2025-06-13

**Authors:** Andrés Camargo, Rafael Vargas, Alexander Rincón-Rodríguez, Elena Jiménez, Martha Trujillo-Güiza

**Affiliations:** 1 PhD Program in Health Sciences, School of Medicine, Universidad Antonio Nariño, Bogotá 111511, Colombia; 2 School of Health and Sport Sciences, Fundación Universitaria del Área Andina, Bogotá 111221, Colombia; 3 Facultad de Medicina y Ciencias de la Salud, Universidad Militar Nueva Granada (UMNG), Bogotá 110111, Colombia; rvargas3200@hotmail.com; 4 Secretariat of Health, Alcaldia Municipal de Tocancipá, Tocancipá 251010, Colombia; saludpsicologiatocancipa@gmail.com; 5 School of Nursing, Universidad Mariana, Pasto 520001, Colombia; mejimenez@umariana.edu.co; 6 Facultad de Medicina, Universidad Antonio Nariño, Ibagué 730001, Colombia

**Keywords:** resilience, armed conflict, psychosocial factors, social support, Colombia

## Abstract

Background: This study examines the psychosocial factors influencing resilience in Colombian victims of armed conflict, highlighting the role of personal, family, and community resources in mitigating trauma. Resilience is a dynamic process that enables individuals and communities to adapt to adversity. Given Colombia’s prolonged violence and forced displacement, trauma has significantly impacted both physical and emotional health. Methods: 200 adult conflict victims were recruited through snowball sampling and completed validated scales via a secure online platform. The Connor–Davidson Resilience Scale (CD-RISC-10), the APGAR Family Scale, and the Brief Resilience Coping Scale assessed resilience, social support, and psychological well-being. Results: Findings indicate that family support was strongly associated with higher resilience, with participants exhibiting higher family support scores (mean = 15.6, SD = 4.47) demonstrating significantly greater resilience (*p* < 0.001). Additionally, resilient coping strategies (Exp(B) = 0.772, *p* < 0.001) and higher subjective happiness (Exp(B) = 0.864, *p* = 0.001) were identified as key predictors of resilience. Significant correlations were found between resilience and mental health outcomes, with higher resilience linked to lower anxiety (ρ = −0.388, *p* < 0.001) and depression (ρ = −0.388, *p* < 0.001). Education, employment, and socioeconomic status also positively influenced resilience, with individuals with higher educational levels (χ^2^ = 21.265, *p* = 0.006) and income (χ^2^ = 8.945, *p* = 0.030) showing higher resilience scores. In contrast, alcohol consumption (Exp(B) = 0.813, *p* = 0.014) was negatively associated with resilience. Conclusions: This study shows that resilience in conflict victims is influenced by both individual and social factors. Strengthening family and community support, along with improving coping strategies, is essential for long-term recovery, highlighting the need for targeted interventions to enhance psychosocial well-being in affected populations.

## 1. Introduction

The impact of violence on the mental health of victims of armed conflict extends beyond the immediate effects of traumatic events. In the Colombian context, prolonged exposure to violence and the conditions of forced displacement create what is known as “cumulative trauma”, where victims experience a constant cycle of stress that affects their emotional and physical well-being (Bell et al., 2012; Restrepo & Padilla-Medina, 2023). This cumulative trauma not only affects individuals’ ability to process and overcome traumatic events but can also perpetuate a cycle of poverty and vulnerability, exacerbating the socio-economic difficulties faced by conflict victims. Furthermore, the lack of adequate access to mental health services in many regions affected by the conflict worsens psychological disorders, preventing individuals from receiving the necessary treatment for recovery (Gómez-Restrepo et al., 2016; Bonilla-Escobar et al., 2021).

In this sense, efforts to address the psychosocial consequences of the conflict must go beyond treating the individual symptoms of trauma and focus on creating programs that strengthen the resilience of affected communities. Recent research suggests that community interventions, including social support and rebuilding of family and community support networks, are crucial in assisting the recovery of armed conflict victims. These interventions not only allow for psychological recovery but also help restore the social fabric of communities that have been severely damaged by violence and displacement. Therefore, an integrated approach that addresses both individual and collective needs is crucial to mitigate the effects of the conflict and promote true long-term healing for war victims.

Resilience emerges as a crucial factor in the recovery process for victims of armed conflict, as it not only enables the overcoming of immediate trauma effects but also facilitates long-term adaptation in contexts of prolonged violence and forced displacement. Rather than focusing solely on the symptoms of trauma, resilience is understood as the capacity of individuals and communities to adapt positively to adversities, overcoming the emotional and physical difficulties arising from violence (Bonanno, 2004; Masten, 2014). However, resilience is not an innate characteristic; it is a dynamic process influenced by a complex interaction of personal, family, and community factors that act as protective resources against the impact of traumatic events (Adams & Boscarino, 2006; Benight & Bandura, 2004; Bonanno, 2004; Masten, 2014).

Strengthening resilience involves recognizing the importance of social and family support networks. Individuals who maintain such networks during and after conflict show higher resilience levels, which is key in countering the chronic stress and psychological effects of cumulative trauma (Gómez-Restrepo et al., 2016). Access to community resources and fostering cooperation among community members can also significantly reduce symptoms of mental disorders, such as PTSD and depression, which are common among conflict-affected populations (Tol et al., 2011). In this sense, resilience should be understood as an individual process and a collective phenomenon that depends on the restoration of social bonds and collaboration in rebuilding the community (Oviedo et al., 2022).

This paper addresses how these factors interrelate and affect the resilience of armed conflict victims. At the individual level, characteristics such as emotional control, adaptability to change, and the ability to manage stress and adversity are evaluated, which are fundamental for resilience (Gómez-Restrepo et al., 2023; Herrera-Moreno et al., 2018). At the family level, emotional support and the quality of family relationships are crucial for the well-being of the victims. Furthermore, at the community level, access to support networks and social resources has been identified as a protective factor against the effects of trauma. Community interventions that promote solidarity and cooperation among community members are essential for reducing the impact of mental disorders resulting from conflict (Gómez-Restrepo et al., 2016).

The aim of this paper is to identify and analyze the psychosocial factors influencing resilience in a sample of victims of armed conflict in Colombia, evaluating the impact of individual, family, and community variables to provide evidence that supports the design of effective psychosocial interventions to strengthen resilience and facilitate the comprehensive recovery of the victims. Based on these gaps in the literature, this study aims to test the following hypothesis:

Higher levels of family support, resilient coping strategies, and subjective happiness will be positively associated with greater resilience in Colombian victims of armed conflict, with sociodemographic factors such as education and socioeconomic status also acting as significant predictors.

## 2. Materials and Methods

Recruitment and Sampling: A total of 200 Colombian adults (mean age = 34.2 ± 9.5 years, range: 18–65) recognized as victims by government agencies (Colombia’s Registro Único de Víctimas (RUV)) were included in the study. Participants were recruited via snowball sampling through social leaders and victims affiliated with both governmental and non-governmental organizations. The sample included individuals from a broad range of municipalities across Colombia, with significant representation from regions affected by the armed conflict, such as Cundinamarca, Bogotá, and Nariño. Snowball sampling allowed for the inclusion of participants from both urban and rural areas, ensuring a diverse and comprehensive sample. Exclusion criteria included individuals aged 65 years or older and those with neurological disorders or cognitive impairments. The survey was conducted using a secure web platform, ensuring anonymity and compliance with international data protection standards. Participation was entirely voluntary, with no financial incentives provided for participation or for referring others. Informed consent was obtained from all participants, ensuring they were fully aware of the study’s purpose, procedures, and their rights, including voluntary participation and confidentiality.

### 2.1. Data Collection

The database was conducted through a secure online platform supplied by Sinopsis Servicio y Soluciones SAS, version 1.0. ensuring anonymity and security of responses. The platform was optimized for mobile devices to facilitate participation from individuals in remote areas, ensuring inclusivity and accessibility. Participants completed validated scales via this platform in both self-administered and hetero-administered format, further optimized for mobile accessibility.

### 2.2. Measures

Participants completed validated scales via a secure online platform (Sinopsis Servicio y Soluciones SAS) in self-administered and hetero-administered formats optimized for mobile accessibility. The battery included:

Sociodemographic, Mental Health Survey, and Exposure to Armed Conflict: This survey assesses data such as age, sex, marital status, educational level, occupation, income, types of violence experienced, and displacement reasons based on the official RUV categories. It ensured data consistency and validity by following recognized classifications of victimization (e.g., physical violence, sexual violence, forced disappearance, homicide, kidnapping, extortion, and forced displacement). The questionnaire also collected information on access to mental health services and neuropsychiatric history, both personal and familial.

### 2.3. Mental Health Assessment

#### 2.3.1. Connor–Davidson Resilience Scale (CD-RISC 10)

The Connor–Davidson Resilience Scale (CD-RISC-10) is a widely used instrument for measuring resilience, defined as the ability to recover from adversity. This 10-item Likert scale asks participants to indicate how much they agree with each statement. The items assess factors such as emotional control, adaptability to change, confidence in one’s abilities, and the capacity to recover from stress or adversity. The CD-RISC-10 has shown strong reliability and validity across various populations, including those exposed to traumatic events (Bakic & Ajdukovic, 2019; Notario-Pacheco et al., 2014). Higher scores on the scale indicate greater resilience, and it is commonly used to categorize participants into resilient and non-resilient groups. Typically, a score of 30 or higher suggests a high level of resilience, while lower scores indicate lower levels of resilience. This cut-off point has been validated in previous studies (Campbell-Sills & Stein, 2007; Connor & Davidson, 2003). The CD-RISC-10 is favored for its brevity, high reliability, and strong psychometric properties in clinical and research settings. Regarding reliability, the Cronbach’s alpha for this scale is 0.839, indicating very good internal consistency, suggesting that the items are highly correlated and provide reliable measurements of resilience.

#### 2.3.2. Brief Resilience Coping Scale (BRCS)

The Brief Resilience Coping Scale (BRCS) is a 4-item Likert scale designed to measure how individuals cope with stress and adversity in a resilient manner. It has shown high internal consistency, with a Cronbach’s alpha greater than 0.70, indicating reliable measurements of resilient coping. Higher scores on the scale reflect better resilience. The BRCS has been validated across various cultural contexts, including Latin America, and it effectively assesses stress management, emotional regulation, and positive attitudes in individuals of different ages (Limonero et al., 2014; Sinclair & Wallston, 2004; Tomas et al., 2021). Additionally, the BRCS has demonstrated strong reliability in Spanish-speaking cancer patients, with an omega value of 0.86, confirming its effectiveness as a reliable tool for measuring resilient coping in both general and clinical populations (Calderón et al., 2022).

#### 2.3.3. The APGAR Family Scale

The APGAR is a Likert scale that measures family functioning across five key dimensions: adaptation, participation, affection, growth, and resolution. It is commonly used to assess the level of family support and the quality of family relationships (Arias, 1994; Smilkstein, 1978; Smilkstein et al., 1982). In Colombia, the APGAR scale has been adapted for health studies and has been widely used (Arias, 1994; Díaz-Cárdenas et al., 2017). Moreover, a Colombian version was developed, which includes two additional questions about social support networks to assess the social support from friends, providing a more comprehensive evaluation of social support (Arias, 1994). The scale has demonstrated very high reliability, with a Cronbach’s alpha of 0.926, indicating excellent internal consistency. Regarding the interpretation of scores, the scale ranges from 0 to 20, with higher scores indicating better family functioning. A score between 0–9 is associated with severe family dysfunction, 4–7 with moderate dysfunction, 14–17 with mild dysfunction, and 18–20 suggests good family functioning (Arias, 1994; Smilkstein et al., 1982). Furthermore, a combined score from both the APGAR Family and APGAR Friends scales (maximum score of 28) can be used to provide a comprehensive assessment of social and family support.

#### 2.3.4. AUDIT-C Questionnaire

The AUDIT-C Questionnaire is a 3-item tool designed to assess alcohol consumption patterns and identify risky drinking behaviors. It is widely used in both clinical and research settings to detect harmful alcohol consumption (Babor et al., 2001). The scale has demonstrated acceptable reliability, with a Cronbach’s alpha of 0.763, indicating moderate internal consistency. The item statistics showed a mean of 1.85 and a standard deviation of 2.357. Higher scores on the scale indicate greater risk of problematic alcohol consumption. A score of 4 or higher typically indicates risky drinking behavior, which may require further assessment or intervention. This cut-off is widely used in clinical and research contexts to identify individuals at risk for alcohol-related issues (Babor et al., 2001).

#### 2.3.5. Generalized Anxiety Disorder-2 (GAD-2) and Patient Health Questionnaire-2 (PHQ-2)

The GAD-2 and PHQ-2 are ultra-short scales designed to rapidly identify symptoms of anxiety and depression, respectively. These scales have shown good reliability and validity in quickly detecting individuals who may need further assessment for anxiety or depression (Kroenke et al., 2003; Löwe et al., 2005), including those from Spanish-speaking populations (Errazuriz et al., 2022). The Cronbach’s alpha for the GAD-2 scale is 0.860, indicating high reliability, which suggests excellent internal consistency among the items of the scale. A score of 3 or higher on the GAD-2 is typically considered indicative of clinically significant anxiety symptoms, warranting further evaluation. For the PHQ-2, the Cronbach’s alpha is 0.783, indicating acceptable reliability, with moderate internal consistency. A score of 3 or higher on the PHQ-2 usually points to clinically significant depressive symptoms, requiring further assessment or intervention (Kroenke et al., 2003). Both scales are valuable tools for the quick screening of anxiety and depression symptoms, providing a reliable means of identifying individuals who may require more in-depth evaluation (Errazuriz et al., 2022; Kroenke et al., 2003; Löwe et al., 2005; Plummer et al., 2016).

#### 2.3.6. Posttraumatic Stress Disorder (PTSD)-8

The PTSD-8 is a brief screening tool designed to assess core symptoms of Post-Traumatic Stress Disorder (PTSD), including re-experiencing, avoidance, and hyperarousal. It has demonstrated strong psychometric properties in various trauma-exposed populations, such as disaster victims, assault survivors, and displaced individuals (Andersen et al., 2018; Forrest & Steel, 2023; Hansen et al., 2010). For the interpretation of the PTSD-8, a score of 3 or higher on at least one item in each of the three symptom groups—intrusion, avoidance, and hyperarousal—indicates the presence of clinically significant PTSD symptoms (Andersen et al., 2018). The scale is widely used in clinical and research settings to detect PTSD across diverse populations exposed to traumatic events. The Cronbach’s alpha for the subscales are as follows: 0.871 for sub-avoidance (excellent reliability), 0.808 for sub-intrusion (good reliability), and 0.766 for sub-hyperactivity (acceptable reliability). These reliability measures indicate strong internal consistency, making the PTSD-8 an effective tool for screening PTSD symptoms.

#### 2.3.7. Subjective Happiness Scale (SHS)

The Subjective Happiness Scale (SHS) is a 4-item instrument designed to assess individuals’ overall perception of their happiness and life satisfaction, based on their self-assessment of happiness relative to others. This scale measures subjective well-being and has demonstrated high reliability and validity across various cultural contexts, including Latin America and Ibero-America (Lyubomirsky & Lepper, 1999; Extremera & Fernández-Berrocal, 2014). Due to its strong psychometric properties, the SHS is widely used in cross-cultural research on happiness.

In terms of reliability, the SHS presents a Cronbach’s alpha of 0.778, indicating good internal consistency. Higher scores reflect greater well-being and life satisfaction, indicating a higher level of subjective happiness (Lyubomirsky & Lepper, 1999). The scale is generally used to categorize scores into low, moderate, and high subjective happiness levels based on participants’ responses, with higher scores suggesting a greater sense of happiness and well-being.

### 2.4. Statistical Analysis

The data were analyzed using SPSS v.28. Descriptive statistics were used to calculate means and standard deviations for all variables. The Shapiro–Wilk test was used to evaluate the normality distribution, and skewness and kurtosis were checked for further distribution analysis. Pearson correlation was used to assess associations between psychosocial variables.

A Multivariate Analysis of Variance (MANOVA) evaluated the differences between resilient and non-resilient groups. Post-hoc Tukey tests and effect sizes were calculated to interpret significant differences.

A Chi-Square (χ^2^) test was used to assess the relationship between resilience and various sociodemographic factors, including sex, education level, income, and region of residence. Significant associations were found between these factors and resilience.

A logistic regression analysis was conducted to identify predictors of resilience. Variables such as gender, age, time since displacement, and depressive symptoms were analyzed to predict the likelihood of being classified as resilient. Multicollinearity was assessed using the Variance Inflation Factor (VIF), and significant predictors were selected through a stepwise regression approach.

To classify participants as resilient or non-resilient, a cut-off score of 30 was used on the Connor–Davidson Resilience Scale (CD-RISC-10), based on previous studies (Campbell-Sills & Stein, 2007; Connor & Davidson, 2003). Participants with scores above 30 were classified as resilient, while those with scores below 30 were categorized as non-resilient. This cut-off point has been validated in the literature as a reliable method for distinguishing between high and low levels of resilience in populations exposed to trauma.

## 3. Results

### 3.1. Sample Characteristics

#### 3.1.1. Sociodemographic Descriptive Data

The sample consisted of 200 Colombian adults recognized as victims of armed conflict, according to the Registro Único de Víctimas (RUV). The mean age of participants was 34.2 years (SD = 9.5, range: 18–65 years). Regarding sex distribution, 62% of the sample were female. A statistically significant difference was found in resilience distribution by sex (χ^2^ = 6.263, *p* = 0.012), with a higher proportion of men classified as resilient compared to women.

Sociodemographic characteristics also included 58% of participants identified as displaced persons, and 42% reported multiple forms of victimization (e.g., forced displacement, homicide of relatives).

Regarding educational level, 34.5% of participants had completed secondary education, 20.5% had technical studies, and 12% had incomplete primary education. A significant relationship was found between educational level and resilience (χ^2^ = 21.265, *p* = 0.006), indicating that participants with lower educational attainment were less likely to be resilient.

Regarding employment status, most participants were employed (41.5%) or unemployed (22.5%). However, no significant differences were found between employment status and resilience (χ^2^ = 5.924, *p* = 0.314). Monthly income distribution showed that 69% of participants reported earning less than the Colombian minimum wage (approximately USD 280 per month at the time of the study), and just 0.5% earned more than six times that amount (USD 1680 or more). A significant relationship was found between income level and resilience (χ^2^ = 8.945, *p* = 0.030), with a higher proportion of non-resilient individuals in the lower-income groups.

In terms of geographic distribution, most participants resided in Cundinamarca (40.0%), followed by Bogotá (17.5%) and Nariño (13.0%). Residence in certain regions showed a significant relationship with resilience (χ^2^ = 30.896, *p* = 0.030), highlighting that participants in Nariño had the lowest resilience levels.

#### 3.1.2. Bivariate Analysis

Bivariate analyses were conducted to explore the relationships between sociodemographic factors and resilience, as well as to analyze associations between other psychological variables. Spearman’s correlation coefficients indicated significant relationships between age and resilience (ρ = 0.178, *p* = 0.012), and between socioeconomic factors (e.g., income level, education) and resilience. The results suggest that higher education and income levels were associated with greater resilience, while lower-income groups were predominantly non-resilient.

Additionally, in relation to psychological variables, correlations between resilience, subjective happiness, and anxiety were also analyzed. Resilient individuals reported significantly higher levels of subjective happiness and family support (measured by the APGAR scale), while they exhibited lower levels of anxiety and depression.

#### 3.1.3. Factors Related to Armed Conflict Exposure

The primary reasons for displacement reported by participants were armed confrontations (37.0%) and direct threats (27.0%). However, no significant relationship was found between the reason for displacement and resilience (*p* = 0.178).

Regarding the type of violence experienced, 54.7% of resilient individuals and 49.0% of non-resilient individuals reported having suffered forced displacement. Other forms of violence included the homicide of a family member (17.0%), physical violence (9.5%), and the forced disappearance of a family member (6.0%). No significant association was found between the type of violence experienced and resilience (*p* = 0.349).

Regarding socioeconomic classification, the Sistema de Identificación de Potenciales Beneficiarios de Programas Sociales (SISBEN) was used to assess participants’ levels of socioeconomic vulnerability. A significant relationship was found between poverty level and resilience (χ^2^ = 20.293, *p* = 0.000). Non-resilient individuals were more frequently classified in the extreme poverty category (41.5%), whereas resilient individuals were more evenly distributed across less vulnerable categories.

### 3.2. Normality Tests

Kolmogorov–Smirnov and Shapiro–Wilk normality tests indicated that most variables did not follow a normal distribution (*p* < 0.05), except for resilience (*p* = 0.200 and *p* = 0.156, respectively) (Table 1 and Table S1). Therefore, non-parametric tests were used in subsequent statistical analyses.

### 3.3. Group Differences (Resilient vs. Non-Resilient Individuals)

Analysis of group differences between resilient and non-resilient individuals revealed several significant variables. Resilience was significantly associated with sex, educational level, income, place of residence, anxiety, depression, subjective happiness, and family support (APGAR). There are no significant differences concerning age, marital status, employment, or APGAR Friends. (Tables S2–S4).

### 3.4. Multivariate Analysis (MANOVA)

The MANOVA analysis showed a significant effect of the resilience group on multiple psychological variables, as detailed in Table 2 and Table S6. The analysis indicated that resilient individuals exhibited significantly higher levels of family support, subjective happiness, and lower levels of anxiety and depression compared to non-resilient individuals. Specifically, Pillai’s Trace was F(4, 315) = 0.1697, with a *p*-value < 0.001, and a partial η^2^ = 0.170, indicating a moderate effect size.

The MANOVA results indicate that resilient individuals reported significantly higher scores on APGAR, reflecting better family support, and higher levels of subjective happiness (M = 21.51) compared to non-resilient individuals (M = 16.98). Resilient individuals also had significantly lower levels of anxiety (M = 1.21) and depression (M = 1.09) compared to non-resilient individuals (M = 2.16 and M = 1.90, respectively). The F-values for these differences were significant, with *p*-values all below 0.001.

#### Correlation Heatmap of Key Psychosocial Variables

**Figure 1:**
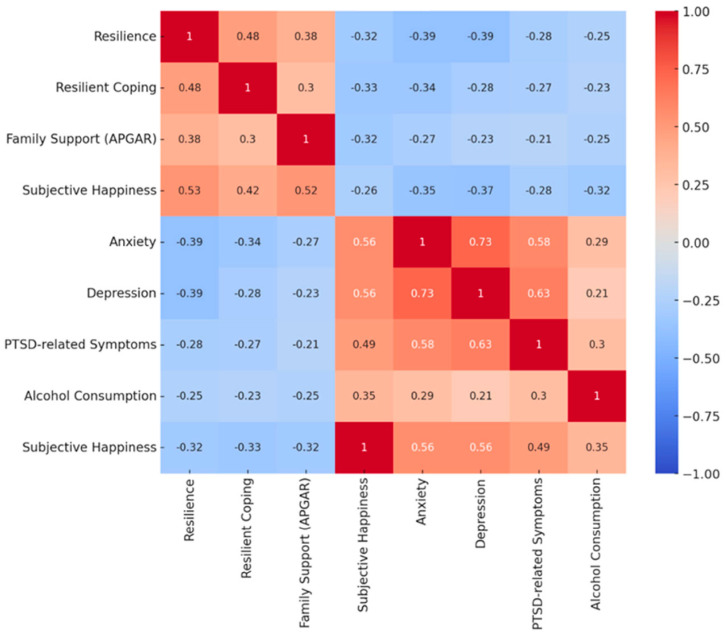
Heatmap with Pearson correlation coefficients between key psychosocial variables: Correlation values close to +1 indicate strong positive relationships, while values near −1 reflect strong negative relationships. A value of 0 means no correlation. Note: The color scale represents the strength and direction of the correlation: red indicates a strong positive correlation (close to +1), blue means a strong negative correlation (close to −1), and white indicates no correlation (0). The significance levels for each correlation coefficient are provided in Table S5, which adds further context to the relationships shown in this heatmap.

The Spearman correlation coefficients between key psychosocial variables—resilience, coping strategies, family support (measured by APGAR), subjective well-being, anxiety, depression, PTSD-related symptoms, and alcohol consumption—are presented in a heatmap (Figure 1). Notably, strong positive correlations were found between anxiety and depression (0.73), anxiety and PTSD-related symptoms (0.58), and depression and PTSD-related symptoms (0.63). Additionally, anxiety and subjective happiness showed a negative correlation of −0.56, indicating that higher levels of anxiety were associated with lower subjective happiness.

Further details of these correlations are available in Table S5, which provides the Spearman correlation coefficients for the full sample, as well as separately for the resilient and non-resilient groups.

### 3.5. Predictive Models of Resilience

The binary logistic regression model identified four significant predictors of resilience (Table 3). These predictors were analyzed based on their odds ratios (Exp(B)) and *p*-values, providing a clear understanding of how each variable influences resilience.

#### Interpretation of Odds Ratios, Model Fit, and Predictive Ability

The odds ratios (Exp(B)) from the binary logistic regression model indicated that a one-unit increase in resilient coping increased the odds of being resilient by 22.8% (Exp(B) = 0.772). Similarly, a one-unit increase in subjective happiness was associated with a reduction of 13.6% in the odds of being classified as non-resilient (Exp(B) = 0.864). Conversely, higher levels of anxiety increased the odds of being classified as non-resilient by 44.7% (Exp(B) = 1.447), while higher levels of alcohol consumption decreased the odds of resilience by 18.7% (Exp(B) = 0.813).

The overall logistic regression model demonstrated good fit and predictive capacity. The Omnibus Tests showed significant improvement at each step (*p* < 0.001), indicating that each predictor significantly contributed to the model. The model accounted for between 25.3% (Cox & Snell R^2^) and 36.9% (Nagelkerke R^2^) of the variability in resilience. Additionally, the Hosmer–Lemeshow Test confirmed an acceptable fit (*p* > 0.05).

Overall classification accuracy was 79.0%, indicating strong predictive performance, especially in correctly identifying non-resilient individuals. However, the model was less precise (47.2%) in classifying resilient individuals, suggesting the need for further refinement. (Additional details of the logistic regression model are provided in Supplementary Table S7).

## 4. Discussion

International research on resilience has explored various populations affected by conflicts, such as expatriates, refugees, children in war zones, and civilians in armed conflict situations. These studies highlight protective factors and coping strategies across diverse sociopolitical contexts, providing valuable insights into how individuals cope with and overcome adversities. These findings emphasize the importance of contextual factors, including the sociopolitical environment and social networks, in shaping resilience (Meyer et al., 2019; Oviedo et al., 2022; Purgato et al., 2020). In Latin America, research has predominantly focused on mental health and resilience in vulnerable communities, emphasizing cultural elements, social support, and historical memory as crucial factors in overcoming adversity. The regional approach underscores the significance of sociocultural contexts in resilience development (Vera-Bachmann, 2015; Zamora-Moncayo et al., 2021). In Colombia, research has targeted specific groups, such as women victims of sexual violence, adolescents, displaced adults, and families affected by armed conflict, but a comprehensive analysis considering both psychosocial and demographic variables has been lacking (Miller-Suchet et al., 2024; Perdomo et al., 2021). This gap limits our understanding of resilience’s full impact and hinders the design of effective interventions. This study addresses this gap by providing a broad perspective on resilience in Colombian armed conflict victims, complementing previous studies with a focus on specific aspects.

The findings of this study reaffirm prior research highlighting the critical role of social support and family structure in fostering resilience. In particular, the study found that family functionality, as measured by the APGAR Family Scale, positively correlates with resilience. Families with high functionality, characterized by strong communication and mutual support, provide a nurturing environment that enhances the ability to cope with trauma. Previous research corroborates these findings, emphasizing the importance of family cohesion in facilitating trauma recovery (Nam et al., 2016; Özmete & Pak, 2023). These findings underline the pivotal role of family dynamics in supporting individuals exposed to extreme trauma, highlighting that families provide essential resources for resilience in adverse circumstances.

Regarding demographic factors, this study found that age, gender, geographic origin, and socioeconomic status significantly influenced resilience. Older participants demonstrated higher resilience, suggesting that accumulated life experience and coping skills enhance the ability to manage trauma (Aldwin, 2009; Cosco et al., 2017). This is consistent with findings in the literature on resilient aging, where older adults, despite facing more challenges, should adapt positively by utilizing available resources. In terms of gender, men displayed higher resilience levels than women, aligning with studies suggesting that women tend to face great difficulties in developing resilience, particularly in contexts of emotional abuse (Watters et al., 2021; Wei et al., 2021). The study also found that geographic origin played a role in resilience, with participants from Cesar and Meta showing higher levels of resilience versus those from Nariño. This may be attributed to stronger community support networks and social cohesion, which have been identified as key factors in resilience (Nuwayhid et al., 2011; Saghin et al., 2022).

Moreover, socioeconomic factors such as education and employment were positively associated with resilience. Those in extreme poverty showed lower resilience levels, reinforcing the importance of economic stability and access to resources in fostering resilience (Wong et al., 2023; Weitzel et al., 2023). The study found that individuals with higher educational levels and stable employment exhibited greater resilience, suggesting that resources such as income and education contribute to adaptive coping strategies, ultimately improving resilience in the face of adversity.

While this study did not find a statistically significant correlation between access to mental health services and resilience, it highlighted the importance of social support and the availability of resources in determining resilient responses. This is consistent with the literature that emphasizes the role of mental health services in promoting flexible coping strategies (Bonanno & Westphal, 2024). Although direct evidence linking mental health service access to resilience was not found, the results underscore the need for accessible mental health care to support the development of adaptive coping mechanisms among trauma survivors.

Regarding psychosocial factors, the study found that resilience was positively correlated with resilient coping, family support (APGAR), and subjective happiness. These factors act as protective elements against the development of mental health disorders, such as PTSD and depression, confirming that resilient coping and strong social networks are crucial for mitigating the impact of trauma (Bonanno, 2004). Conversely, symptoms of anxiety, depression, and PTSD, particularly with characteristics of intrusion, avoidance, and hyperactivity, were negatively correlated with resilience, indicating that these symptoms act as barriers to resilience and emotional recovery (Blanc et al., 2016). The negative correlation between alcohol consumption and resilience suggests that individuals with higher alcohol consumption levels are less resilient, and alcohol use may exacerbate mental health problems, further reducing resilience (Cusack et al., 2023; Nielsen & Andersen, 2022). These findings support the idea that interventions aimed at strengthening resilience should focus on reducing symptoms of anxiety, depression, and PTSD, as these represent significant obstacles to developing resilience.

Finally, the study supports the dynamic nature of resilience, as proposed by systems theory. Resilience is not a fixed state but an adaptive process influenced by various factors. This perspective highlights the need for continuous support and interventions that help individuals maintain resilience over time. Resilience fluctuates in response to social support, resilient coping, and subjective well-being, factors that can act as buffers or triggers when facing trauma (Scheffer et al., 2024). This reinforces the importance of addressing mental health symptoms dynamically and fostering continuous resources that support long-term resilience and adaptation in conflict-affected populations.

Limitations: This study has several limitations. First, the 10-item Connor–Davidson Resilience Scale (CD-RISC-10), though widely used, is a brief measure and may not capture all aspects of resilience. Second, while a mix of self-reported and hetero-administered data was used, biases associated with self-reporting may still be present. The use of snowball sampling may also limit the representativeness of the sample. Additionally, although regional differences were explored, the findings may not be generalizable to all regions of Colombia. Lastly, no significant correlation was found between access to mental health services and resilience, highlighting the need for further research in this area. Despite these limitations, the study offers valuable insights into resilience in conflict-affected populations.

## 5. Conclusions

This paper provides valuable insights into the psychosocial factors influencing resilience among Colombian victims of armed conflict. The findings highlight that resilience is not solely an individual trait, but rather a dynamic process shaped by family support, effective coping strategies, and social networks. Strong family functionality, as measured by the APGAR Family Scale, and the use of resilient coping strategies were key predictors of higher resilience in this population. These findings suggest that interventions aimed at enhancing these psychosocial resources could significantly improve recovery outcomes for conflict-affected individuals.

The study also emphasizes the importance of socio-demographic factors such as education, income, and gender in shaping resilience. Older participants and those with higher education and stable employment levels showed higher resilience, indicating that socio-economic stability plays a critical role in adaptive coping strategies. These findings point to the need for tailored interventions that address not only individual mental health needs but also family dynamics and community support systems.

In addition, the study found that mental health challenges, particularly symptoms of PTSD, depression, and anxiety, are significant barriers to resilience. The negative correlations between these symptoms and resilience suggest that therapeutic interventions should focus on reducing these barriers to enhance recovery. Furthermore, the study underscores the need for improving access to mental health services in conflict-affected regions, as mental health care plays a crucial role in facilitating the development of resilient coping mechanisms.

Finally, this study highlights the dynamic nature of resilience, which fluctuates in response to ongoing social support, resilient coping, and psychological well-being. A comprehensive approach that includes individual, family, and community-level interventions is essential for promoting long-term recovery and adaptation in populations affected by conflict. Future research should focus on regional differences within Colombia to better understand how local contexts influence resilience and tailor interventions accordingly.

By addressing these psychosocial determinants, public health policies and interventions can better support the long-term recovery of armed conflict victims, helping to break the cycle of trauma and violence in Colombia.

## Figures and Tables

**Table 1:** Normality test results for resilience, psychological well-being, and social support variables.

Variable	Kolmogorov–Smirnov (*p*-Value)	Shapiro–Wilk (*p*-Value)
Resilience	0.200	0.156
APGAR family support	0.003	0.002
Anxiety	0.000	0.000
Depression	0.000	0.000
Subjective happiness	0.001	0.001

**Table 2:** Results of the MANOVA for psychological and family support variables in resilient and non-resilient individuals.

Variable	Resilient (M ± SD)	Non-Resilient (M ± SD)	F-Value	*p*-Value	η^2^ Parcial
APGAR	15.60 ± 4.469	12.10 ± 5.434	17.749	<0.001	0.082
Anxiety	1.21 ± 1.419	2.16 ± 1.771	12.518	0.001	0.059
Depression	1.09 ± 1.535	1.90 ± 1.538	10.644	0.001	0.051
Subjective Happiness	21.51 ± 4.685	16.98 ± 5.603	27.649	<0.001	0.123

**Table 3:** Results of the binary logistic regression model for predictors of resilience.

Predictor	Exp(B)	*p*-Value
Resilient coping	0.772	<0.001
Subjective happiness	0.864	0.001
Anxiety	1.447	0.010
Alcohol consumption	0.813	0.014

## Data Availability

The data supporting the results of this study will be available upon request to the corresponding author. Due to privacy, legal, or ethical restrictions, the data are not publicly available, but the reason for these restrictions will be provided. Contact information will be provided so researchers can request access to the data.

## Supplementary Materials

The following supporting information can be downloaded at: https://www.mdpi.com/article/10.3390/bs15060816/s1, Table S1: Results of Normality Tests (Kolmogorov-Smirnov and Shapiro-Wilk) for Psychosocial Variables; Table S2: Results of Cross-tabulations between Sociodemographic Variables and Resilience Groups; Table S3: Results of Cross-tabulations between Socioeconomic Variables, Variables Related to the Conflict, and Resilience Groups; Table S4: Results of Cross-tabulations between Variables Related to Access to Mental Health Services and Support, and Resilience Groups; Table S5: Spearman Correlations Between Psychosocial Variables in the Full Sample, Resilient Group, and Non-Resilient Group; Table S6: Multivariate Analysis of Variance for Psychosocial Variables between Resilient and Non-Resilient Groups in Armed Conflict Victim Population; Table S7: Binary Logistic Regression for Predictors of Resilience in Armed Conflict Victims in Colombia.
