# Supplementary material for: Psychosocial Factors Influencing Resilience in a Sample of Victims of Armed Conflict in Colombia: A Quantitative Study
Source: Behav Sci (Basel). 2025 Jun 13;15(6):816. doi: 10.3390/bs15060816 (PMC12190149; doi:10.3390/bs15060816)
Supplement: Supplementary file 1 [file behavsci-15-00816-s001.zip › behavsci-3525076-supplementary.pdf]

Psychosocial Factors Influencing Resilience in a Sample of Victims of Armed Conflict in Colombia: A Quantitative Study

Table S1. Results of Normality Tests (Kolmogorov-Smirnov and Shapiro-Wilk) for Psychosocial Variables

**Variable**	**Kolmogorov-Smirnov (p-value))**	**Shapiro-Wilk (p-value)**	**Normality**
Resilience	0.200	0.156	Normal
Resilient Coping	0.001	0.009	Non-normal
APGAR	0.000	0.000	Non-normal
APGAR Friends	0.000	0.000	Non-normal
Alcohol Consumption	0.000	0.000	Non-normal
Anxiety	0.000	0.000	Non-normal
Depression	0.000	0.000	Non-normal
Subjective Happiness	0.001	0.001	Non-normal
Sub-avoidance	0.000	0.000	Non-normal
Sub-intrusion	0.000	0.000	Non-normal
Sub-hyperactivity	0.000	0.000	Non-normal

Cross-tabulations

Table S2. Results of Cross-tabulations between Sociodemographic Variables and Resilience Groups

Variable	Category	Resilient (n = 53)	Non-Resilient (n = 147)	Total (N = 200)	χ^2	p
**Sex**	Female	27 (20.8%)	103 (79.2%)	130 (65.0%)	6.263	0.012
	Male	26 (37.1%)	44 (62.9%)	70 (35.0%)		
**Marital Status**	Single	23 (23.2%)	76 (76.8%)	99 (49.5%)	1.532	0.675
	Married	26 (29.5%)	62 (70.5%)	88 (44.0%)		
	Divorced	3 (27.3%)	8 (72.7%)	11 (5.5%)		
	Widowed	1 (50.0%)	1 (50.0%)	2 (1.0%)		
**Educational Level**	Technical Studies	13 (31.7%)	28 (68.3%)	41 (20.5%)	21.265	0.006
	Incomplete University Studies	3 (27.3%)	8 (72.7%)	11 (5.5%)		
	Completed University Studies	4 (66.7%)	2 (33.3%)	6 (3.0%)		
	Postgraduate	6 (75.0%)	2 (25.0%)	8 (4.0%)		
	Completed Primary	2 (13.3%)	13 (86.7%)	15 (7.5%)		
	Incomplete Primary	3 (12.5%)	21 (87.5%)	24 (12.0%)		
	Incomplete Completed Primary	17 (24.6%)	52 (75.4%)	69 (34.5%)		
	Completed Secondary	5 (25.0%)	15 (75.0%)	20 (10.0%)		
	Incomplete Secondary	0 (0.0%)	6 (100.0%)	6 (3.0%)		
**Occupation**	Employed	27 (32.5%)	56 (67.5%)	83 (41.5%)	5.924	0.314
	Student	2 (8.7%)	21 (91.3%)	23 (11.5%)		
	Self-employed	8 (29.6%)	19 (70.4%)	27 (13.5%)		
	Retired	1 (33.3%)	2 (66.7%)	3 (1.5%)		
	Homemaker	5 (26.3%)	14 (73.7%)	19 (9.5%)		
	Unemployed	10 (22.2%)	35 (77.8%)	45 (22.5%)		
**Age**	Mean (SD)	37.68 (11.35)	35.91 (11.68)	36.38 (11.59)		0.354
**Children**	Mean (SD)	1.75 (1.62)	1.99 (1.68)	1.94 (1.66)		0.295

Note: The χ^2 values correspond to the Pearson Chi-square test for each variable and the Mann-Whitney U test for age and number of children. Proportions are presented in percentage (%).

Table S3. Results of Cross-tabulations between Socioeconomic Variables, Variables Related to the Conflict, and Resilience Groups

Variable	Category	Resilient (n = 53)	Non-Resilient (n = 147)	Total (N = 200)	χ^2	p
**Approximate Monthly Income**	More than 6 SMMLV	1 (100.0%)	0 (0.0%)	1 (0.5%)	8.945	0.030
(SMMLV = USD 280)	From 2 to 4 SMMLV	3 (42.9%)	4 (57.1%)	7 (3.5%)		
	From 1 to 2 SMMLV	20 (37.0%)	34 (63.0%)	54 (27.0%)		
	Less than 1 SMMLV	29 (21.0%)	109 (79.0%)	138 (69.0%)		
**Current Residence**	Cundinamarca	23 (28.8%)	57 (71.3%)	80 (40.0%)	30.896	0.030
(Regions/Provinces)	Bogotá	11 (31.4%)	24 (68.6%)	35 (17.5%)		
	Nariño	4 (15.4%)	22 (84.6%)	26 (13.0%)		
	Valle del Cauca	2 (13.3%)	13 (86.7%)	15 (7.5%)		
	Boyacá	2 (22.2%)	7 (77.8%)	9 (4.5%)		
	Putumayo	0 (0.0%)	5 (100.0%)	5 (2.5%)		
	Caldas	0 (0.0%)	4 (100.0%)	4 (2.0%)		
	Meta	2 (66.7%)	1 (33.3%)	3 (1.5%)		
	Cesar	3 (100.0%)	0 (0.0%)	3 (1.5%)		
	Vichada	1 (33.3%)	2 (66.7%)	3 (1.5%)		
	Cauca	0 (0.0%)	3 (100.0%)	3 (1.5%)		
	Arauca	1 (50.0%)	1 (50.0%)	2 (1.0%)		
	Tolima	1 (100.0%)	0 (0.0%)	1 (0.5%)		
	Santander	1 (50.0%)	1 (50.0%)	2 (1.0%)		
	Sucre	2 (100.0%)	0 (0.0%)	2 (1.0%)		
	Huila	0 (0.0%)	2 (100.0%)	2 (1.0%)		
	Caquetá	0 (0.0%)	2 (100.0%)	2 (1.0%)		
	Antioquia	0 (0.0%)	1 (100.0%)	1 (0.5%)		
	Amazonas	0 (0.0%)	2 (100.0%)	2 (1.0%)		
**Main Cause of Displacement**	Armed combat or confrontations	17 (32.1%)	57 (38.8%)	74 (37.0%)	6.3	0.178
	Direct threats	20 (37.0%)	34 (63.0%)	54 (27.0%)		
	Mass displacement	11 (20.8%)	27 (18.4%)	38 (19.0%)		
	Forced recruitmet	3 (5.7%)	21 (14.3%)	24 (12.0%)		
	Extreme poverty	2 (3.8%)	8 (5.4%)	10 (5.0%)		
**Type of Violence Suffered**	Displacement	29 (54.7%)	72 (49.0%)	101 (50.5%)	7.814	0.349
	Murder of a family member	8 (15.1%)	26 (17.7%)	34 (17.0%)		
	Physical violence	2 (3.8%)	17 (11.6%)	19 (9.5%)		
	Forced disappearance of a family member	6 (11.3%)	6 (4.1%)	12 (6.0%)		
	Other	4 (7.5%)	9 (6.1%)	13 (6.5%)		
	Extortion	3 (5.7%)	9 (6.1%)	12 (6.0%)		
	Sexual violence	1 (1.9%)	5 (3.4%)	6 (3.0%)		
	Kidnapping	0 (0.0%)	3 (2.0%)	3 (1.5%)		

**SISBEN Classification**	Group A (extreme poverty)	9 (17.0%)	61 (41.5%)	70 (35.0%)	20.293	0.000
	Group B (moderate poverty)	14 (26.4%)	38 (25.9%)	52 (26.0%)		
	Group C (Vulnerability)	12 (22.6%)	27 (18.4%)	39 (19.5%)		
	Group D (not poor, not vulnerable)	6 (11.3%)	14 (9.5%)	20 (10.0%)		
	Not classified	12 (22.6%)	7 (4.8%)	19 (9.5%)		

Note: The χ^2 values correspond to the Pearson Chi-square test for each variable. Proportions are presented in percentage (%).

Table S4. Results of Cross-tabulations between Variables Related to Access to Mental Health Services and Support, and Resilience Groups

Variable	Category	Resilient (n = 53)	Non-Resilient (n = 147)	Total (N = 200)	χ^2	p
**Government or NGO Support Post-Displacement**	No	24.3% (42)	75.7% (131)	86.5% (173)	3.25	0.071
	Yes	40.7% (11)	59.3% (16)	13.5% (27)		
**Access to Mental Health Services Post-Displacement**	No	28.9% (48)	71.1% (118)	83.0% (166)	2.92	0.087
	Yes	14.7% (5)	85.3% (29)	17.0% (34)		
**Family History of Neuropsychiatric Disorders (Diagnosed by Psychiatrist)**	None	34.3% (46)	65.7% (88)	67.0% (134)	15.25	0.004
	Mood disorders (major depression, bipolar disorder)	8.1% (3)	91.9% (34)	18.5% (37)		
	Anxiety disorders (generalized anxiety disorder, panic disorder, obsessive-compulsive disorder)	9.1% (2)	90.9% (20)	11.0% (22)		
	Substance use disorders (alcoholism, drug use)	40.0% (2)	60.0% (3)	2.5% (5)		
	Personality disorders (borderline personality disorder, antisocial personality disorder)	0.0% (0)	100.0% (2)	1.0% (2)		
**Personal History of Neuropsychiatric Disorders (Diagnosed by Psychiatrist)**	None	31.2% (43)	68.8% (95)	69.0% (138)	6.44	0.168
	Mood disorders (major depression, bipolar disorder)	10.3% (3)	89.7% (26)	14.5% (29)		
	Anxiety disorders (generalized anxiety disorder, panic disorder, obsessive-compulsive disorder)	23.8% (5)	76.2% (16)	10.5% (21)		

Substance use disorders (alcoholism, drug use)	20.0% (2)	80.0% (8)	5.0% (10)
Personality disorders (borderline personality disorder, antisocial personality disorder)	0.0% (0)	100.0% (2)	1.0% (2)

Note: The χ^2 values correspond to the Pearson Chi-square test for each variable. Proportions are presented in percentage (%).

Table S5. Spearman Correlations Between Psychosocial Variables in the Full Sample, Resilient Group, and Non-Resilient Group

Variable	Age	Years of Exposure to Conflict	Resilience	Resilient Coping	APGAR	APGAR Friends	Alcohol Consumption	Anxiety	Depression	Avoidance	Intrusion	Hyperactivity	Subjective Happiness
**Total Sample (N = 200)**													
Age	1	-0.058	0.178*	0.109	0.006	-0.063	-0.076	-0.207**	-0.221**	-0.103	-0.104	-0.160*	0.091
Years of Exposure to Conflict	-0.058	1	-0.073	-0.122	-0.087	-0.014	0.170*	0.064	0.086	0.007	0.04	0.037	-0.144*
Resilience	0.178*	-0.073	1	0.478**	0.384**	0.150*	-0.016	-0.388**	-0.388**	-0.279**	-0.242**	-0.116	0.528**
Resilient Coping	0.109	-0.122	0.478**	1	0.409**	0.254**	-0.089	-0.201**	-0.251**	-0.088	-0.048	-0.131	0.319**
APGAR	0.006	-0.087	0.384**	0.409**	1	0.515**	-0.192**	-0.324**	-0.309**	-0.115	0.021	-0.088	0.503**
APGAR Friends	-0.063	-0.014	0.150*	0.254**	0.515**	1	-0.104	-0.135	-0.159*	0.048	0.1	0.088	0.316**
Alcohol Consumption	-0.076	0.170*	-0.016	-0.089	-0.192**	-0.104	1	0.173*	0.198**	0.176*	0.117	0.1	-0.252**
Anxiety	-0.207**	0.064	-0.388**	-0.201**	-0.324**	-0.135	0.173*	1	0.727**	0.473**	0.301**	0.291**	-0.394**
Depression	-0.221**	0.086	-0.388**	-0.251**	-0.309**	-0.159*	0.198**	0.727**	1	0.449**	0.279**	0.265**	-0.424**
Avoidance	-0.103	0.007	-0.279**	-0.088	-0.115	0.048	0.176*	0.473**	0.449**	1	0.496**	0.428**	-0.259**
Intrusion	-0.104	0.04	-0.242**	-0.048	-0.088	0.1	0.117	0.301**	0.279**	0.496**	1	0.325**	-0.241**
Hyperactivity	-0.160*	0.037	-0.116	-0.131	0.021	0.088	0.1	0.291**	0.265**	0.325**	1	-0.135	0.057
Subjective Happiness	0.091	-0.144*	0.528**	0.319**	0.503**	0.316**	-0.252**	-0.394**	-0.424**	-0.259**	-0.241**	-0.135	1

Variable	Age	Years of Exposure to Conflict	Resilience	Resilient Coping	APGAR	APGAR Friends	Alcohol Consumption	Anxiety	Depression	Avoidance	Intrusion	Hyperactivity	Subjective Happiness
**Resilient Group (N = 53)**													
Age	1	-0.071	0.055	0.035	0.141	0.028	-0.128	-0.394**	-0.456**	-0.021	0.07	-0.23	-0.215
Years of Exposure to Conflict	-0.071	1	0.003	-0.086	-0.202	0.046	-0.028	0.21	0.051	-0.002	-0.058	0.094	0.006
Resilience	0.055	0.003	1	0.331*	0.042	0.294*	0.007	0.006	-0.23	-0.05	0.183	-0.057	0.171
Resilient Coping	0.035	-0.086	0.331*	1	0.251	0.234	-0.061	0.039	-0.09	0.233	-0.021	0.099	
APGAR	0.141	-0.202	0.042	0.251	1	0.427**	-0.155	-0.041	-0.043	0.111	0.062	0.267	0.232
APGAR Friends	0.028	0.046	0.294*	0.234	0.427**	1	0.134	0.126	-0.044	0.163	0.147	0.24	0.203
Alcohol Consumption	-0.128	-0.028	0.007	-0.061	-0.155	0.134	1	0.177	0.132	0.146	0.092	0.387**	-0.280*
Anxiety	-0.394**	0.21	0.006	0.039	-0.041	0.126	0.177	1	0.573**	0.115	0.486**	-0.238	
Depression	-0.456**	0.051	-0.23	-0.09	-0.043	-0.044	0.132	0.573**	1	-0.043	0.317*	-0.329*	
Avoidance	-0.021	-0.002	-0.05	0.233	0.111	0.163	0.146	0.434**	0.406**	1	0.152	0.410**	-0.047
Intrusion	0.07	-0.058	0.183	0.317*	0.062	0.147	0.092	0.115	-0.043	0.152	1	0.339*	-0.064
Hyperactivity	-0.23	0.094	-0.057	-0.021	0.267	0.24	0.387**	0.486**	0.317*	0.339*	1	-0.056	
Subjective Happiness	-0.215	0.006	0.171	0.099	0.232	0.203	-0.280*	-0.238	-0.329*	-0.056	-0.064	-0.056	1

Variable	Age	Years of Exposure to Conflict	Resilience	Resilient Coping	APGAR	APGAR Friends	Alcohol Consumption	Anxiety	Depression	Avoidance	Intrusion	Hyperactivity	Subjective Happiness
**Non-Resilient Group (N = 147)**													
Age	1	-0.047	0.218**	0.071	-0.069	-0.102	-0.071	-0.152	-0.113	-0.105	-0.145	-0.132	0.14
Years of Exposure to Conflict	-0.047	1	-0.07	-0.104	-0.06	-0.036	0.247**	0.001	0.103	0.002	0.052	-0.182*	0.017
Resilience	0.218**	-0.07	1	0.340**	0.286**	0.085	-0.097	-0.331**	-0.322**	-0.314**	-0.261**	-0.151	0.492**
Resilient Coping	0.071	-0.104	0.340**	1	0.402**	0.275**	-0.132	-0.138	-0.179*	-0.115	-0.072	-0.119	0.302**
APGAR	-0.069	-0.06	0.286**	0.402**	1	0.541**	-0.248**	-0.356**	-0.305**	-0.131	-0.078	-0.039	0.522**
APGAR Friends	-0.102	-0.036	0.085	0.275**	0.541**	1	-0.217**	-0.202*	-0.179*	0.034	0.108	0.038	0.310**
Alcohol Consumption	-0.071	0.247**	-0.097	-0.132	-0.248**	-0.217**	1	0.225**	0.254**	0.197*	0.137	-0.007	-0.318**
Anxiety	-0.152	0.001	-0.331**	-0.138	-0.356**	-0.202*	0.225**	1	0.727**	0.475**	0.311**	0.209*	-0.358**
Depression	-0.113	0.103	-0.322**	-0.179*	-0.305**	-0.179*	0.254**	0.727**	1	0.454**	0.341**	0.239**	-0.390**
Avoidance	-0.105	0.002	-0.314**	-0.115	-0.131	0.034	0.197*	0.475**	0.454**	1	0.547**	0.434**	-0.277**
Intrusion	-0.145	0.052	-0.261**	-0.072	-0.078	0.108	0.137	0.311**	0.341**	0.547**	1	0.321**	-0.248**
Hyperactivity	-0.132	0.017	-0.151	-0.119	-0.039	0.038	-0.007	0.209*	0.239**	0.321**	1	-0.171*	-0.171*
Subjective Happiness	0.14	0.017	0.492**	0.302**	0.522**	0.310**	-0.318**	-0.358**	-0.390**	-0.277**	-0.248**	-0.171*	1

***Note*. The Spearman correlation coefficients (ρ) are presented.

*p < .05; **p < .01.**

Tabla S6. Multivariate Analysis of Variance for Psychosocial Variables between Resilient and Non-Resilient Groups in Armed Conflict Victim Population

**Variable**	**Resilient (M ± SD)**	**Non-Resilient (M ± SD)**	**F**	**p**	**Partial η²**
APGAR	15.60 ± 4.469	12.10 ± 5.434	17.749	0.000	0.082
APGAR Friends	4.91 ± 2.364	4.35 ± 2.204	2.408	0.122	0.012
Anxiety	1.21 ± 1.419	2.16 ± 1.771	12.518	0.001	0.059
Depression	1.09 ± 1.535	1.90 ± 1.538	10.644	0.001	0.051
Sub-intrusion	0.09 ± 0.295	0.21 ± 0.409	3.614	0.059	0.018
Sub- hyperactivity	0.19 ± 0.395	0.22 ± 0.419	0.293	0.589	0.001
Sub_ avoidance	0.08 ± 0.267	0.18 ± 0.383	3.16	0.077	0.016
Subjective Happiness	21.51 ± 4.685	16.98 ± 5.603	27.649	0.000	0.123

Note: M = Mean; SD = Standard Deviation; F = F statistic for the effect of resilience; Partial η^2 = Partial eta squared.

Table S7. Binary Logistic Regression for Predictors of Resilience in Armed Conflict Victims in Colombia

Variable	B (Coefficient)	S.E.	Wald	p	Exp(B)	95% CI for Exp(B)
Constant	6.117	1.151	28.257	0.000	453.483	
Resilient Coping	-0.259	0.065	16.014	0.000	0.772	0.680 - 0.876
Alcohol Consumption	-0.207	0.084	6.100	0.014	0.813	0.689 - 0.958
Anxiety	0.369	0.143	6.687	0.010	1.447	1.094 - 1.915
Subjective Happiness	-0.146	0.044	11.034	0.001	0.864	0.793 - 0.942

Note: Exp(B) represents the odds ratio (OR).
